# Optimized Pt–Co Alloy Nanoparticles for Reverse Water–Gas Shift Activation of CO_2_

**DOI:** 10.1021/acsanm.4c00111

**Published:** 2024-04-24

**Authors:** Ákos Szamosvölgyi, Ádám Pitó, Anastasiia Efremova, Kornélia Baán, Bence Kutus, Mutyala Suresh, András Sápi, Imre Szenti, János Kiss, Tamás Kolonits, Zsolt Fogarassy, Béla Pécz, Ákos Kukovecz, Zoltán Kónya

**Affiliations:** † Interdisciplinary Excellence Centre, Department of Applied and Environmental Chemistry, University of Szeged, Rerrich Béla tér 1, Szeged H-6720, Hungary; ‡ Department of Molecular and Analytical Chemistry, University of Szeged, Dóm tér 7−8, Szeged H-6720, Hungary; § HUN-REN-SZTE Reaction Kinetics and Surface Chemistry Research Group, Szeged,H-6720, Hungary; ∥ HUN-REN Centre for Energy Research, Institute of Technical Physics and Materials Science, Budapest H-1121, Hungary

**Keywords:** Pt, Co, alloy nanoparticles, reverse water−gas shift reaction, carbon monoxide

## Abstract

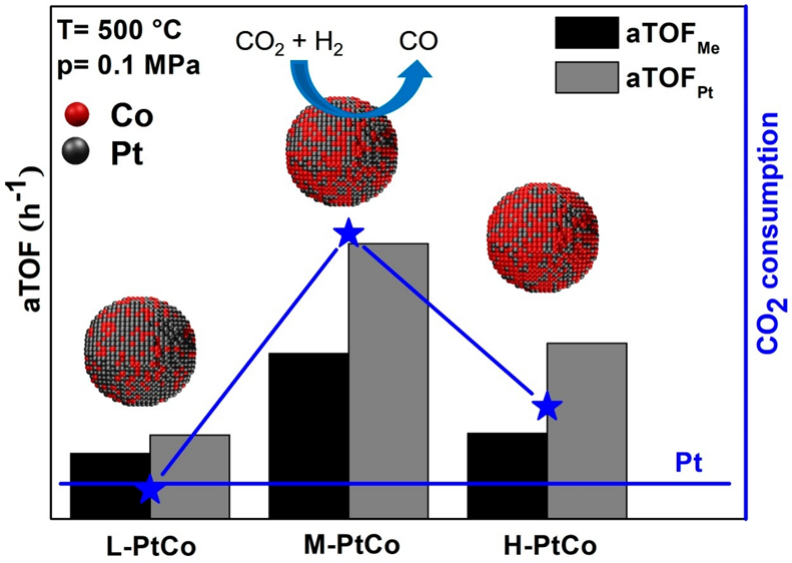

Different Co contents were used to tune bimetallic Pt–Co nanoparticles with a diameter of 8 nm, resulting in Pt:Co ratios of 3.54, 1.51, and 0.96. These nanoparticles were then applied to the MCF-17 mesoporous silica support. The synthesized materials were characterized with HR-TEM, HAADF-TEM, EDX, XRD, BET, ICP-MS, *in situ* DRIFTS, and *quasi in situ* XPS techniques. The catalysts were tested in a thermally induced reverse water–gas shift reaction (CO_2_:H_2_ = 1:4) at atmospheric pressure in the 200–700 °C temperature range. All bimetallic Pt–Co particles outperformed the pure Pt benchmark catalyst. The nanoparticles with a Pt:Co ratio of 1.51 exhibited 2.6 times higher activity and increased CO selectivity by 4% at 500 °C. Experiments proved that the electron accumulation and alloying effect on the Pt–Co particles are stronger with higher Co ratios. The production of CO followed the formate reaction pathway on all catalysts due to the face-centered-cubic structure, which is similar to the Pt benchmark. It is concluded that the enhanced properties of Co culminate at a Pt:Co ratio of 1.51 because decreasing the ratio to 0.96 results in lower activity despite having more Co atoms available for the electronic interaction, resulting in the lack of electron-rich Pt sites.

## 1 Introduction

Global warming is a significant environmental issue caused by the high concentration of CO_2_ in the atmosphere. This increase in CO_2_ concentration is mainly due to human activities such as fossil fuel consumption, mining, construction, and the growing automobile and petrochemical industries.^1^ The stability of CO_2_ is the reason for its accumulation in the atmosphere. However, its concentration can be regulated through various methods, such as adsorption and separation and conversion into chemicals and fuels. Additionally, it could also be used as a C1 building block for feedstock materials in the chemical industry.^2,3^ One method of CO_2_ utilization is through a reverse water–gas shift (RWGS) reaction (CO_2_ + H_2_ ⇌ CO + H_2_O; Δ*H*_298 K_ = 41.1 kJ mol^–1^), which converts CO_2_ into CO and H_2_O in an endothermic reaction. The produced CO can be further modified with the Fischer–Tropsch process [*n*CO + (2*n* + 1)H_2_ → C_*n*_H_2*n*+2_ + H_2_O; Δ*H*_298 K_ = −165 kJ mol^–1^], the most significant industrial application. Hydrogenation of CO_2_ can also form CH_4_ via the Sabatier reaction (CO_2_ + 4H_2_ ⇌ CH_4_ + 2H_2_O; Δ*H*_298 K_ = −165 kJ mol^–1^), as a side reaction to the RWGS process. Thermodynamically, the production of CH_4_ is favorable under low-temperature conditions (<300 °C) and high pressure (1–30 bar).^4^ Regardless of the desired products, catalysts are required to break the bonds of CO_2_, and the development of economically, chemically, and environmentally viable catalysts for CO_2_ conversion remains a highly researched topic. There are many reports on the RWGS reaction using metals like Cu, Ni, Fe, and Co as dopants in a mixed transition-metal oxide or as impregnated particles on various support materials like Al_2_O_3_ or CeO_2_.^5−9^

Mono- and bimetallic nanoparticles with different electronic and morphologic structures play a very important role in heterogeneous catalysis reviewed in the recent past.^10^ Pt is one of the most important noble metals that shows remarkable activity and tunable selectivity in heterogeneous catalytic reactions. When bulk Pt(111) is compared to Pt nanoparticles (Pt NPs), the enhanced activity of Pt NPs supported on metal oxides has been attributed to the presence of low-coordinated sites of the particles, the formation of OH functional groups, and the electronic interaction with the oxide support.^11−14^ Panagiotopoulou et al. have studied Pt NPs supported on reducible metal oxides CeO_2_ and TiO_2_ and irreducible metal oxides MgO, Al_2_O_3_, and SiO_2_ for RWGS reaction,^15^ showcasing the diversity of Pt-based catalytic systems. However, not all support materials excel at enhancing a given reaction, as was demonstrated by our research group by comparing three different SiO_2_-based support materials. The MCF-17 mesoporous silica lacks highly concentrated acidic or basic sites compared to Al_2_O_3_ supports for example; also it has an ordered mesostructure with low surface roughness, which, in contrast to SBA-15 or silica foam, reduces the electron density fluctuations in the structure of MCF-17.^16^ These properties result in weak interactions with the loaded nanoparticles, which is useful if characterization of the catalytic properties of the nanoparticles themselves is the goal. Our research group investigated the properties of Pt/SiO_2_, Pt/CoO_*x*_, and Co^0^/CoO_*x*_ systems in ethanol decomposition and RWGS reaction.^16−18^ Pt/CoO_*x*_ systems in the pretreatment of catalysts play the significant role of creating active sites by partial coverage of the particles with Co_*x*_O_*y*_ species or allowing for different reaction pathways such as RWGS and formate for the reaction.^17^ Investigation of Co^0^/CoO_*x*_ systems showed that both phases activate reactants and stabilize intermediates during RWGS reaction or methanation, but the two different forms of Co_3_O_4_ showed different reaction pathways: carboxylate and formate.^18^ Because these metals show interesting behavior and interaction during the RWGS reaction in their pristine or oxide forms, we wanted to extend our knowledge to their alloys. In general, bimetallic nanoparticles have the potential to be exceptional catalysts due to the synergetic effect exhibited by alloying.^19,20^ Pt–Co systems are explored as high-performing catalysts for different reactions like O_2_ reduction reaction, CO oxidation, and water–gas shift reaction^21−23^ and are also effective in the RWGS reaction. Alayoglu et al. have analyzed the properties of PtCo bimetallic particles in the RWGS reaction at high pressure (5.5 bar) and only for a Pt:Co ratio of 1:1. They reported that Pt–Co alloy nanoparticles show a “Pt-like” chemistry in the RWGS reaction, and alloying with Co does not change the mechanism of the reaction.^24^ Different morphologies of Pt_3_Co nanostructures like cubes and octapods were investigated by Khan et al. They concluded that in Pt–Co alloy structures the high negative charge density around Pt atoms plays a key role in increasing the catalytic activity in the RWGS reaction, and by fine-tuning the shape of the nanoparticles, this effect could be amplified.^25^ In alloys with a transition metal and a noble metal, it is also possible that the transition-metal atoms are stabilized by the neighboring noble-metal atoms, preventing the transition metal from oxidizing, resulting in a structure that behaves like the noble metal.^20,26,27^ In extreme cases, binary compounds other than alloys may also show a mimicking behavior, e.g., WC can act as Pt in the isomerization of 2,2-dimethylpropane.^28^ Furthermore, experimenting with Pt–Co alloy nanoparticles with different Pt:Co ratios in the RWGS reaction at atmospheric pressure has not been explored yet in the literature.

In this study, our objective is to investigate how ∼8-nm-diameter Pt and Pt–Co alloy nanoparticles with different Pt:Co ratios (3.54, 1.51, and 0.96) loaded (1 w/w %) onto MCF-17 perform in the RWGS reaction, monitoring their ability to convert CO_2_ and their selectivity toward CO, and explain the differences or lack thereof by identifying the structural characteristics and catalytic active sites.

## 2 Experimental Section

### 2.1 Materials

All analytical-grade chemicals, including chloroplatinic acid (H_2_PtCl_6_·H_2_O), poly(vinylpyrrolidone) (PVP; MW = 40000), cobalt nitrate hexahydrate [Co(NO_3_)_2_·6H_2_O], oleylamine (C_18_H_37_N), tetraethylorthosilicate (TEOS; SiC_8_H_20_O_4_), ethylene glycol (C_2_H_6_O_2_), ethanol (C_2_H_6_O), mesitylene (C_9_H_12_), hydrochloric acid (HCl), hexane (C_6_H_12_), ammonium fluoride (NH_4_F), and acetone (C_3_H_6_O) were purchased from Merck Hungary Ltd. and were used without further purification. For inductively coupled plasma mass spectrometry (ICP-MS) measurements, concentrated HNO_3_ and HCl were used (Aristar for trace metal analysis, VWR Chemicals). Ultrahigh-purity (5.0 quality) gas cylinders of argon, oxygen, nitrogen, hydrogen, and the gas mixture CO_2_:H_2_ = 1:4 were purchased from Messer Hungarogáz Ltd.

### 2.2 Synthesis of Catalysts

#### 2.2.1 Synthesis of Pt NPs

Pt NPs were synthesized with the polyol method.^29^ In a typical synthesis, 80 mg of H_2_PtCl_6_·2H_2_O and 110 mg of PVP were dissolved in 10 mL of ethylene glycol, followed by sonication for 30 min. The mixture was then evacuated in an inert atmosphere to remove moisture and oxygen and then heated at 200 °C for 2 h in an inert Ar atmosphere. The resulting suspension was precipitated with acetone after cooling to room temperature. Pt NPs were obtained by centrifugation, washed with hexane, and stored in 10 mL of ethanol.

#### 2.2.2 Synthesis of Pt–Co Alloy Nanoparticles

To synthesize Pt–Co alloy nanoparticles with three different nominal metal ratios (Pt:Co = 3:1, 1:1, and 1:2), appropriate amounts of H_2_PtCl_6_·2H_2_O and Co(NO_3_)_2_·6H_2_O were dissolved in 5 mL of oleylamine while the solution was heated to 80 °C. Water and other absorbed gases were evacuated from the transparent solution using a rotary vane vacuum pump. The mixture was heated at 230 °C for 2 h, while maintaining an inert Ar atmosphere by bubbling the gas through the system. By the end of the reaction time, the suspension had turned black, indicating the formation of metallic nanoparticles. The product was then precipitated with acetone, separated by centrifugation, washed with hexane, and stored in 10 mL of ethanol. The resulting nanoparticles were denoted as L-PtCo, M-PtCo, and H-PtCo for low (L, Pt:Co = 3:1), medium (M, Pt:Co = 1:1), and high (H, Pt:Co = 1:2) nominal Co loadings, respectively. The actual Pt:Co molar ratios determined by ICP-MS were 3.54 (L-PtCo), 1.51 (M-PtCo), and 0.96 (H-PtCo), respectively (see the details in section 2.3). These values, along with the actual weight fractions of the metals, are presented in Table S1.

#### 2.2.3 Synthesis of the MCF-17 Support

The synthesis of MCF-17 followed the method reported by Schmidt-Winkel et al.^30^ In a polypropylene bottle, 4 g of P123 and 4 g of mesitylene were transferred into a mixture of 10 mL of concentrated HCl and 65 mL of water and then stirred at 40 °C for 2 h. To this solution was added 9.2 mL of TEOS, and the resulting solution was stirred for 10 min followed by aging at the same temperature for 20 h. Afterward, 46 mg of NH_4_F was added and hydrothermally treated at 100 °C for 24 h. The product was collected by filtration, washed with distilled water and ethanol, and dried at 80 °C overnight. The dried compound was calcined at 600 °C for 6 h in static air flow.

#### 2.2.4 Synthesis of MCF-17-Supported Pt and Pt–Co Alloy Nanoparticles

For a given mass of MCF-17, the required volume of Pt and Pt–Co alloy nanoparticle suspensions was added to achieve 1 w/w % metal loading on the MCF-17 support. MCF-17 and the suspension of the nanoparticles were mixed in ethanol, followed by ultrasonication at room temperature for 3 h. The resulting catalysts were obtained by centrifugation, washed with ethanol, and dried at 80 °C for 12 h. The catalysts were labeled Pt/MCF-17, L-PtCo/MCF-17, M-PtCo/MCF-17, and H-PtCo/MCF-17. Catalysts with a 10 wt % loading were also prepared using the same method. This was necessary for the *quasi in situ* XPS measurements because the low metal loading of 1 wt %, which is distributed between the two metals, could not be detected reliably. These samples are labeled as 10-Pt/MCF-17, 10-L-PtCo/MCF-17, 10-M-PtCo/MCF-17, and 10-H-PtCo/MCF-17.

**Figure 1:**
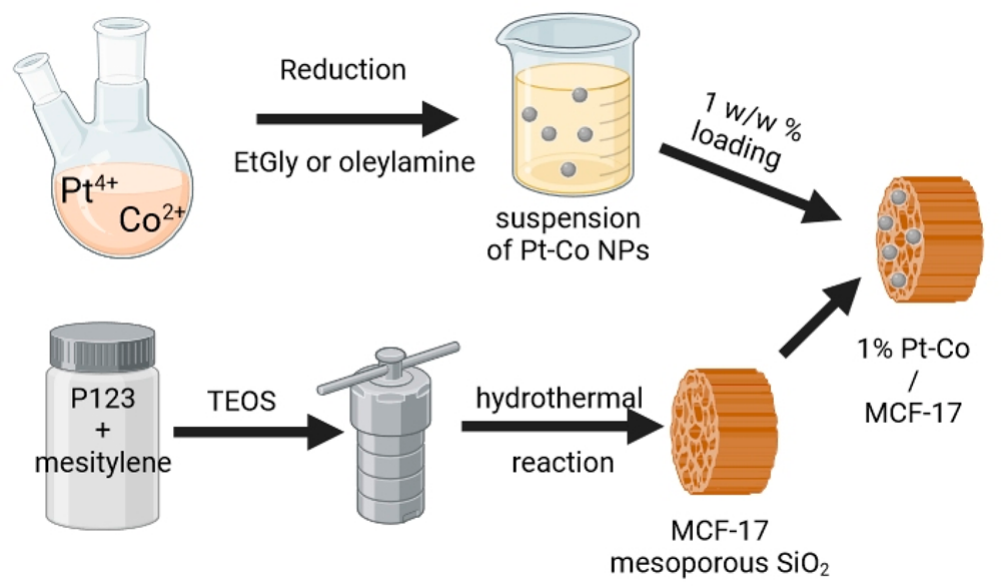
Schematic presentation of catalyst production. Pt or different Pt–Co NPs are synthesized by reducing the metals, while the MCF-17 support material is synthesized separately via a hydrothermal process. A joint suspension of these products is then dried to produce the 1 w/w % Pt–Co NP loaded MCF-17 catalyst materials.

The full process of catalyst production is summarized in Figure 1.

### 2.3 Characterization

A Rigaku Miniflex-II X-ray diffractometer equipped with a Cu Kα X-ray source was used to record X-ray diffraction (XRD) for all synthesized nanoparticles. The nanoparticles were drop-cast onto silica glass for the XRD measurements. A Quantachrome NOVA 3000e gas adsorption analyzer was used to measure N_2_ isotherms at −196 °C. The sample was activated at 200 °C for 2 h under vacuum before the adsorption–desorption isotherms were studied. The specific surface area was calculated based on the Brunauer–Emmett–Teller (BET) theory, and the total pore volume was calculated at a relative pressure of 0.99. Bright-field (BF) TEM images to identify the morphology and particle size distribution were obtained using a FEI TECNAI G2 20 transmission electron microscope operated at a high voltage of 200 kV. An Agilent 7900 inductively coupled plasma torch connected to a mass spectrometer (ICP-MS) was used to determine the Pt and Co content and load of each sample. Here, 10 mg of the catalysts were digested in 5 mL of hot *aqua regia* (50 °C) for 4 h, and then they were filtered, washed, and diluted to 100 mL using deionized water. For quantitation of the elements, the signals of ^59^Co, ^194^Pt, ^195^Pt, and ^196^Pt isotopes were used in addition to the signal of ^88^Y as an internal standard (50 ppb in each sample). High-resolution transmission electron microscopy (HR-TEM), high-angle annular dark-field (HAADF), and energy-dispersive X-ray (EDX) were done in the MFA Thin Film Laboratory, Budapest, Hungary, with a Cs-corrected Themis scanning TEM [(S)TEM] operated with a 200 kV accelerating voltage. EDX mappings were acquired with Super-X EDX detectors in STEM mode.

### 2.4 RWGS Test Reactions

RWGS test reactions were carried out in the fixed-bed reactor from 200 to 700 °C on atmospheric pressure with a gas flow rate of 40 mL min^–1^ (CO_2_:H_2_ = 1:4) using 150 mg of catalyst loaded at the center of the reactor (8 mm i.d.). The catalyst bed, which was typically 2 mm thick, resulted in a gas hourly space velocity (GHSV) of 16000 mL g^–1^ h^–1^. The dead volume of the reactor was filled with quartz beads. The gas line above and below the fixed-bed reactor was heated externally at 150 °C to prevent condensation of the gases. Before the test reactions, the catalysts were oxidized at 300 °C for 30 min using oxygen to remove the PVP or oleylamine capping agent and any other possible contamination from the surface of the catalyst. This was followed by reduction at 300 °C for 1 h using hydrogen gas. The gases in the outlet stream of the reactor were analyzed at regular time intervals using inline gas chromatography (Agilent 6890N gas chromatograph with an HP-PLOT Q column equipped with thermal conductivity and flame ionization detectors). CO_2_ conversion (%) and consumption rate (nmol g^–1^ s^–1^), selectivity of CO, and CH_4_ (%) were calculated using equations reported in the literature:^31^
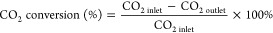

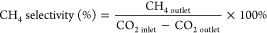

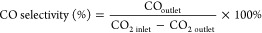
where CO_2 inlet_ and CO_2 outlet_ represent the CO_2_ concentration in the feed and effluent, respectively, and CH_4 outlet_ and CO _outlet_ represent CH_4_ and CO in the effluent, respectively. The catalytic activity is described using a specific apparent turnover frequency (aTOF), defined as the number of CO_2_ molecules converted per hour per Pt (aTOF_Pt_) and per all metal atoms (aTOF_Me_) loaded on the catalyst. The number of loaded atoms is derived from the ICP-MS measurements.

### 2.5 Investigation of the Catalytic Properties

A Kratos XSAM 800 X-ray photoelectron spectroscope was used with *quasi in situ* sample preparation to analyze the effect of pretreatment and reaction conditions. A total of 50 mg of the samples was pressed into 1-cm-diameter circular pellets. The prechamber of the instrument was expanded by a quartz reactor tube, where the pellets were pretreated, and CO_2_ hydrogenation reactions were run. The prechamber was purged with nitrogen and evacuated after pretreatment and reaction. Next, the samples were inserted into the main chamber, and the spectra were collected. To offset the charge accumulation on the sample surface, an electron flood gun was operated during data acquisition. The Pt 4f high-resolution spectra were collected with a pass energy of 40 eV and a step size of 0.1 eV. IR spectroscopy measurements were carried out with an Agilent Cary-670 Fourier transform infrared (FTIR) spectrometer equipped with a Harrick Praying Mantis diffuse-reflectance attachment and two BaF_2_ windows installed in the path of the IR radiation. The spectrometer was purged with nitrogen gas. The spectrum of the pretreated catalyst served as the background for the *in situ* data acquisition. At room temperature, a mixture of CO_2_ and H_2_ with a molar ratio of 1:4 was introduced into the diffuse-reflectance infrared Fourier transform spectroscopy (DRIFTS) cell. The catalyst was heated linearly under the reaction feed from room temperature to 600 °C, with a heating rate of 20 °C min^–1^, and IR spectra were recorded at 100 °C intervals. The spent samples were also investigated with HR-TEM, HAADF, and EDX using the same setup as that described in section 2.3.

## 3 Results and Discussion

### 3.1 Sample Characterization

BF TEM images of the synthesized materials and particle size distribution of Pt–Co and Pt NPs are shown in Figure S1. The average diameter size of Pt NPs was 8.1 ± 1.3 nm. The diameters of the bimetallic Pt–Co NPs were found to be 9.1 ± 2.6, 9.1 ± 2.5, and 7.2 ± 2.0 nm for L-PtCo, M-PtCo, and H-PtCo, respectively. With these average diameters and size distributions, the particle size effect was ruled out as a potential factor for the difference in the catalytic activity. The synthesized nanoparticles were homogeneously distributed on the surface of MCF-17 (Figure S2). HR-TEM images, HAADF-STEM images, and EDX mapping of the bimetallic samples are shown in Figures S3 and2. The nanoparticles Pt, L-PtCo, and M-PtCo H-PtCo have good distribution on the MCF-17 support. EDX mapping indicated that Pt and Co were distributed throughout the bimetallic nanoparticles. L-PtCo shows the most homogeneous distribution of Co, while in the M-PtCo and H-PtCo samples, minor enrichment of the metals has been observed in the HAADF images. The location of these enrichments varies greatly, creating domains where the Pt:Co ratios are different. Some particles exhibit this enrichment in their center; however, they could not be addressed as core–shell particles because Pt and Co are both dispersed.

**Figure 2:**
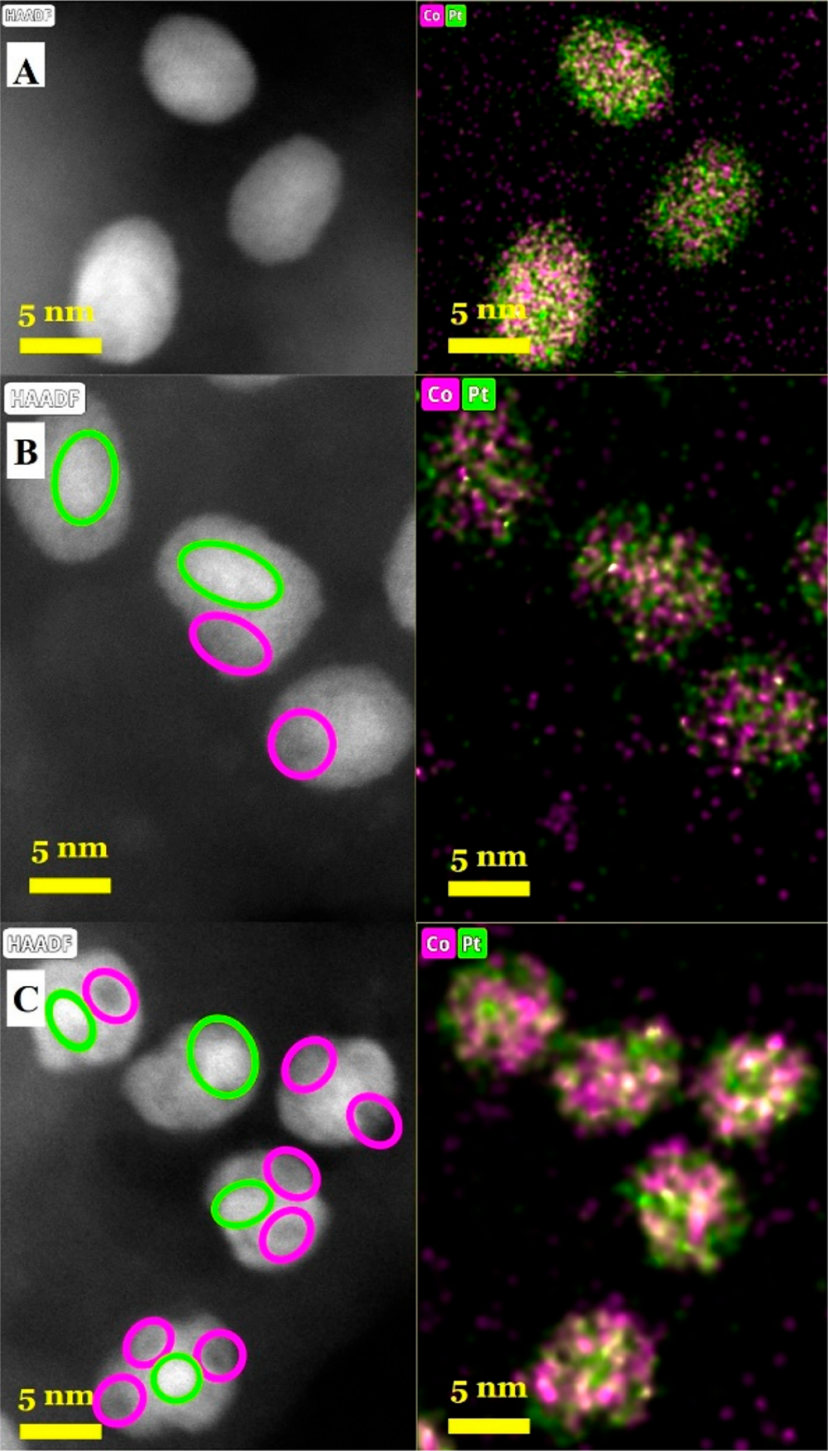
HAADF images and EDX element mapping of the prepared Pt–Co bimetallic catalysts: (A) L-PtCo/MCF-17; (B) M-PtCo/MCF-17; (C) H-PtCo/MCF-17. Minor enrichments of Pt and Co are marked on the HAADF images, with circles matching the colors of the EDX element mapping.

**Figure 3:**
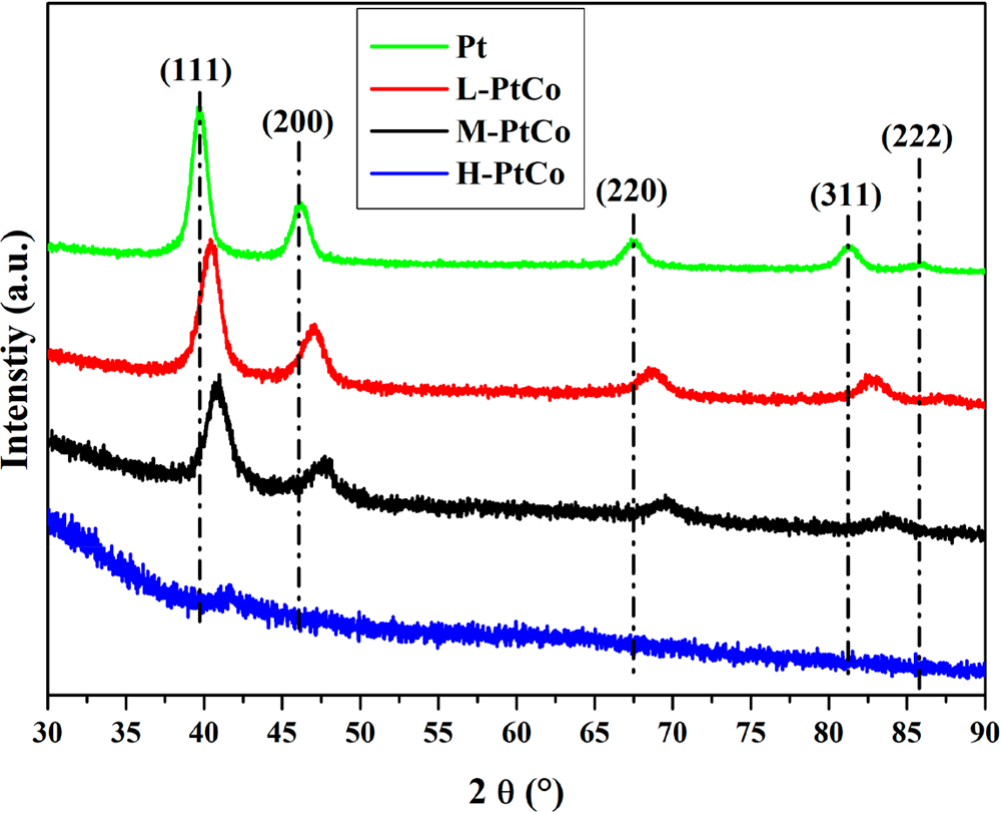
XRD patterns of Pt and Pt–Co alloy nanoparticles drop-cast onto a glass slide.

The synthesized Pt NPs have shown XRD reflections at 2θ = 39.7°, 46.2°, 67.3°, 81.2°, and 85.7° corresponding to the (111), (200), (220), (311), and (222) planes of a face-centered-cubic (fcc) structure, respectively (Figure 3; ICDD PDF 70-2431).^32^ For bimetallic Pt–Co NPs, the diffraction pattern was similar to that of Pt NPs, and no reflections related to Co_3_O_4_ appeared. However, the positions of the reflections shifted to a higher 2θ angle, which confirmed that Co and Pt coexist in the crystal lattice (ICDD PDF 77-7553).^33,34^ Note that the reflection of H-PtCo was analyzed after a Savitzky–Golay smoothing of the data due to significant broadening of the reflections. When the Co ratio is increased, the shift in the 2θ position of the reflections is more prominent, indicating a decrease of the *d* spacing of the fcc lattice (Table 1). This is expected considering the smaller radius of Co atoms (152 pm) compared to that of Pt atoms (177 pm). The reflections are also broadened, indicating that the nanoparticles are polycrystalline in nature and more Co content promotes the formation of smaller primer crystallites within the particles because the nanoparticle sizes are almost uniform across all types of Pt–Co particles, as shown by the TEM images (Figure S1). This feature also gives a plausible explanation for the different Pt and Co enrichments within the particles, shown by the HAADF and EDX images (Figure 2). This difference in the XRD properties is also predicted by theoretical calculations created for bulk stoichiometric Pt, CoPt_3_, and CoPt, confirming that the synthesized samples fit into the trend established by the standards (Figure S4).^35^ According to the phase diagram of Pt and Co based on the experimental and computational methods, Pt–Co systems are prone to forming disordered crystal structures when the stoichiometry is not met,^36^ which should explain the weak reflections of H-PtCo.

**Table 1:** Basic Crystallographic Properties Calculated from Scherrer and Bragg’s Equation Using the Parameters of the (111) Reflection of the fcc Structure

sample	reflection, 2θ (deg)	fwhm, 2θ (deg)	*d*_111_ (pm)	primer crystallite size (nm)
Pt	39.70	1.24	227	6.8
L-PtCo	40.40	1.58	223	5.4
M-PtCo	40.90	1.78	220	4.8
H-PtCo	41.65	1.96	217	4.3

### 3.2 Catalytic Performance

**Figure 4:**
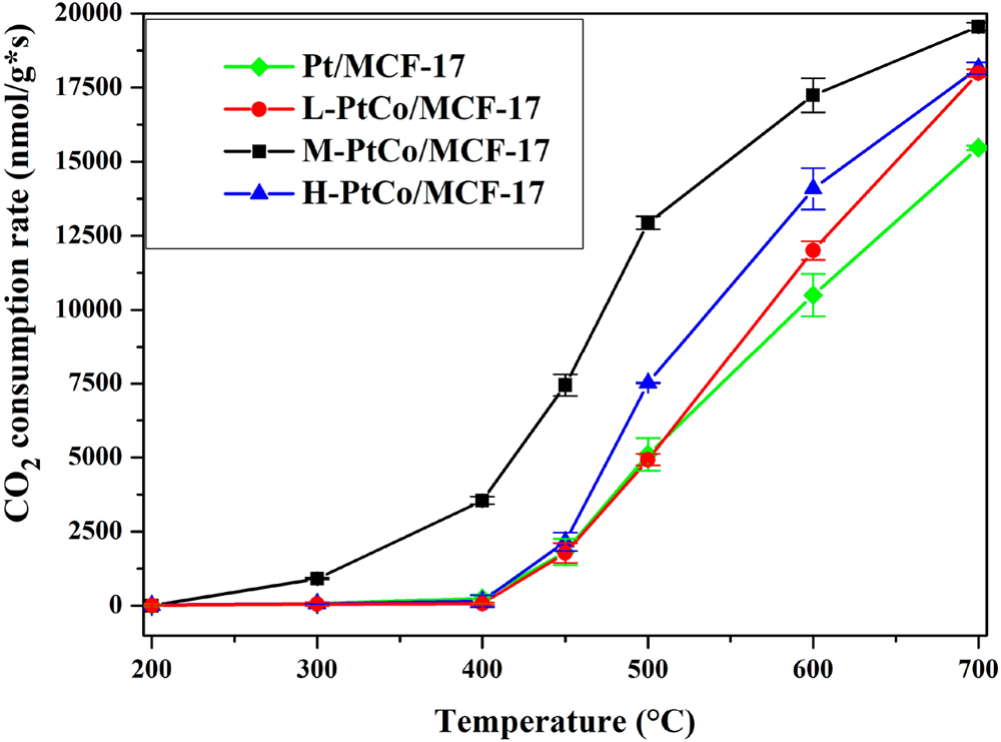
CO_2_ consumption rate of the tested catalytic systems in the 200–700 °C temperature range.

In general, the Pt–Co/MCF-17 catalysts surpassed the pure Pt/MCF-17 system in RWGS test reactions in terms of the CO_2_ consumption rate and conversion of CO_2_ during the process (Figures 4 and S7). The L-PtCo/MCF-17 system showed a better performance than the Pt/MCF-17 system in the high-temperature range from 550 to 700 °C as a result of the enhancing effect of Co in the material.

This effect was increased for M-PtCo/MCF-17, which had noteworthy activity from 300 °C and highly outperformed the other catalysts in the whole temperature range, while Pt/MCF-17, L-PtCo/MCF-17, and H-PtCo/MCF-17 showed CO_2_ conversion of <1% until 450 °C. Further increasing the Co content of the bimetallic nanoparticles, we could not surpass the performance of M-PtCo/MCF-17. H-PtCo/MCF-17 was more active than Pt/MCF-17 and L-PtCo/MCF-17 in the 450–700 °C temperature range, but its activity and conversion rate were significantly lower than the capabilities of M-PtCo/MCF-17. We elucidate the superior catalytic properties of M-PtCo/MCF-17 with the synergetic effect of Pt and Co atoms in the alloy nanoparticle structure. The H-PtCo/MCF-17 catalyst showed diminishing returns on the catalytic activity with increased Co ratio compared to the M-PtCo/MCF-17 catalyst; hence, optimization of the Pt:Co ratio is crucial to the assembly of a highly functional catalyst material. Because of these observed properties, for further experiments (*in situ* DRIFTS and *quasi in situ* XPS), the behavior of the catalysts is highlighted at the 500 °C state, and the CO_2_ consumption rate, CO_2_ conversion, and CO and CH_4_ selectivity for all MCF-17-supported catalysts at 500 °C are presented in Table 2. The catalyst’s lifetime was analyzed for 6 h after reaching 700 °C; during this time, its activity was not impaired (Figure S6).

**Table 2:** CO_2_ Consumption Rate, CO and CH_4_ Selectivity of Pt, and Pt–Co NPs Supported on MCF-17 at 500 °C

catalyst	CO_2_ consumption rate (×10^2^ nmol g^–1^ s^–1^)	CO_2_ conversion (%)	CO selectivity (%)	CH_4_ selectivity (%)
Pt/MCF-17	51	13.3	95.1	4.9
L-PtCo/MCF-17	49	12.6	98.2	1.8
M-PtCo/MCF-17	129	33.5	98.4	1.6
H-PtCo/MCF-17	75	19.8	99.2	0.8

During the RWGS test reactions, two products were detected for all four catalysts, CO and CH_4_; the data are presented in Figures S8 and S9. In the low-temperature range (200–400 °C, CO_2_ conversion of <1%), all catalysts produce CH_4_. At 450 °C and higher temperatures, the production of CO is promoted with high selectivity (>95%). Concerning the selectivity of CH_4_, it significantly decreases with increasing temperature and also decreases upon the addition of Co to the nanoparticles. The Pt/MCF-17 catalyst shows a CH_4_ selectivity of ∼5–6% in the range of 500–700 °C, while it is only <2% in the case of L-PtCo/MCF-17 and <1% for the M-PtCo/MCF-17 and H-PtCo/MCF-17 materials. In Table 3, the samples are compared, specifying their activity for Pt and all metal atoms (sum of Pt and Co) in the catalysts. We list other catalysts for the RWGS reaction from the available literature that contain either Pt or Co or both, and the metals are supported by an irreducible metal oxide or other low-activity support. Where possible, aTOF is also included or calculated from the available data for comprehension of the degree of sum metal and Pt utilization.

**Table 3:** Comparison of CO_2_ Conversion in a RWGS Reaction with Other Pt- or Co-Based Catalysts on Irreducible Metal Oxide Supports Reported in the Literature^a^

catalyst	Pt load (w/w %)	Co load (w/w %)	*T* (°C)	*p* (MPa)	GHSV (mL g^–1^ h^–1^)	aTOF_Pt_ (h^–1^)	aTOF_Me_ (h^–1^)	CO_2_ conversion (%)	CO selectivity (%)	ref
Pt/MCF-17	0.969		500	0.1	16000	370	370	13.3	95.1	this work
L-PtCo/MCF-17	0.701	0.06	500	0.1	16000	491	383	12.6	98.2	this work
M-PtCo/MCF-17	0.562	0.113	500	0.1	16000	1612	968	33.5	98.4	this work
H-PtCo/MCF-17	0.512	0.162	500	0.1	16000	1029	503	19.8	99.2	this work
Pt/SiO_2_	1.67		350	0.1	N/A	N/A	N/A	9.0	99.9	(11)
Pt_50_Co_50_/ MCF-17	3.84	1.16	300	0.55	60000	391	196	5.0	>99.5	(24)
Co/MCF-17		5	300	0.55	60000		91	5.0	82.0	(24)
Pt_3_Co_nanocube_/ active carbon	4.54	0.46	150	3.2	N/A	348	261	N/A	0.0	(25)
Pt_3_Co_octapod_/ active carbon	4.54	0.46	150	3.2	N/A	758	568	N/A	0.0	(25)
Pt/Al_2_O_3_	0.97		500	0.34	12000	2188	2188	33.8	N/A	(37)
Pt/Al_2_O_3_ (commercial)	0.5		500	0.1	12000	358	358	9.7	N/A	(38)
Co/SBA-15		2.6	600	0.1	18000		305	37	100.0	(39)
Na–Co/SBA-15		15	380	1	18000		618	26.5	55.5	(40)
Na–Co/SiO_2_		15	380	1	18000		771	31.2	26.7	(40)
Pt_4_Co_nanowire_/Al_2_O_3_	0.93	0.07	150	3.2		1650	1310	N/A	0.0	(41)
Pt_4_Co_nanowire_/SiO_2_	0.93	0.07	150	3.2		610	484	N/A	0.0	(41)
Pt/SBA-15	0.55		400	0.1	12000	234	234	15.2	98.9	(42)

a The rate of CO_2_ (aTOF) conversion is specified as the number of Pt and all metal atoms for comparison.

The aTOF values can be used as indicators for the utilization of metal atoms, and the values range between 91 and 2188 h^–1^. An aTOF of >1000 h^–1^ usually indicates high conversion and high selectivity for CO_2_. For pure Co systems, higher temperatures are required to reach 100% CO selectivity. The behavior of pure Pt systems is different based on the temperature, GHSV, or pressure, showing the capability of reaching ∼33% conversion in the case of Pt/Al_2_O_3_ if the pressure is increased to 0.34 MPa. These findings further justify that experiments with Pt–Co alloy systems are worth studying and may potentially outperform other catalysts under the same conditions.

### 3.3 *In Situ* DRIFTS

**Figure 5:**
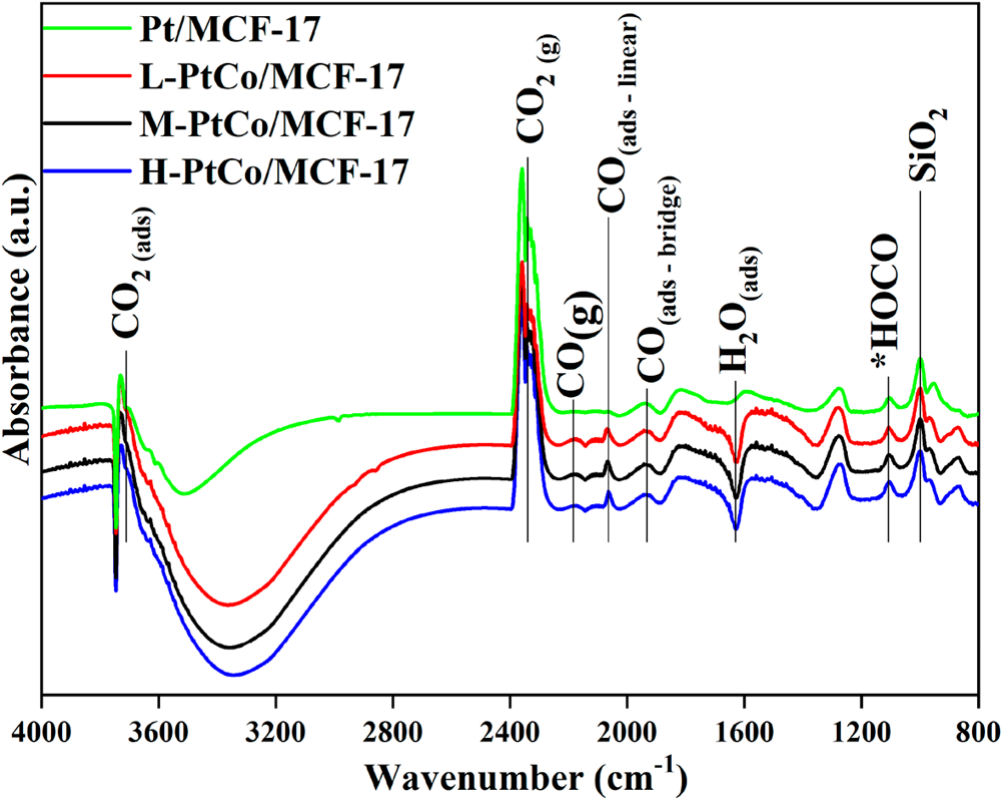
DRIFTS spectra of the catalysts showcasing the intermediates on the sample surface during atmospheric CO_2_ hydrogenation at 500 °C and atmospheric pressure.

The adsorbed surface species and intermediates were investigated during the catalytic test reactions with DRIFTS. Our key finding is that the tested Pt and Pt–Co systems share the same behavior of bonding and activating CO_2_ on their surface in the 200–700 °C temperature range, sharing the same characteristic peaks (Figure S11). Figure 5 shows a comparison of the *in situ* DRIFTS results for the investigated catalysts at 500 °C. The intense bands around 2200 cm^–1^ correspond to gas-phase CO_2_.^17^ Two strong twin bands at 3750–3550 cm^–1^ belong to the combined tones of the gas-phase and adsorbed CO_2_ molecules.^17,18^ Generally, the following reaction steps take place with the activation of CO_2_ and H_2_ during the RWGS reaction in harmony with the density functional theory calculations carried out on Pt(111) and Pt NPs:^11,12^
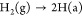
1
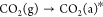
2
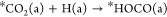
3
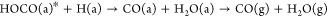
4

In the first step, H_2_ molecules are cleaved on the metallic sites and the generated H atoms react with the CO_2_ adsorbed on the sample surface. This mechanism is also supported by thermodynamic calculations in the case of Pt.^11^ The initial hydrogenation of *CO_2_ to *HOCO is exothermic, and the corresponding *E*_a_ is 1.01 eV. The dissociation of *HOCO to *CO and *OH is also exothermic with an *E*_a_ value of 0.75 eV. The *HOCO intermediate is detected at 1100 cm^–1^. HOCO is hard to detect spectroscopically, due to its short lifetime, especially when reducible oxide is used for support. Recently, this intermediate was observed by high-resolution electron energy loss spectroscopy in a water–gas shift (WGS) reaction (H_2_O + CO ⇌ H_2_ + CO_2_) on Pt_3_Ni(111). Analysis of the vibrational spectrum indicates the formation of HOCO species at 128–131 meV (∼1056 cm^–1^).^43^ Using an *ab initio* molecular dynamics method, this band is located between 1111 and 1011 cm^–1^. It can be suggested that hydrogen-stabilized HOCO in a {HOCOH} adduct has a longer lifetime, so it is detectable more easily by an *in situ* DRIFTS technique.^44^ These steps can go forward in the formate or carboxylate (COOH) pathway. A formate (HCOO) intermediate should be detected around 1570–1590 and 1350 cm^–1^.^16,45−48^ Carboxylate (COOH) frequently appears at ∼1670 and ∼1251 cm^–1^.^17,18,45−47,49^ However, no such peaks were detected; thus, these reaction pathways are suppressed by the lack of suitable supports. Peaks correspond to adsorbed linear CO at 2065 cm^–1^ and bridged CO at 1930 cm^–1^. This CO and also H_2_O are most likely products of further hydrogenation or the decomposition of *HOCO as per step reaction 4. Adsorbed H_2_O is detected as a negative peak at 1625 cm^–1^.^50^ The IR band at ∼1280 cm^–1^ can be derived from two phenomena. It could be an attribute of bidentate or bridge-bonded carbonate as an inactive side product,^45^ or it could indicate the sharp absorption edge characteristic of silica-type materials. Although this feature should be accounted for in the background spectrum, its intensity may change as a function of the temperature and the presence of cospecies.^26^ The observed IR signals around 1800 and around 1000 cm^–1^ and below this wavenumber are attributable also to self-absorption of silica-type supports,^26^ although the bands near 1000 cm^–1^ and somewhat below could be assigned to different carbonites.^45^ The production of methane coming from CO dissociation and the hydrogenation of CO requires the presence of *CH_3_ and *CH_*x*_ fragments, which are further converted into methane. The peaks corresponding to these species appear at 2880–2995 cm^–1^,^51,52^ and Pt/CoO_*x*_ interfaces are required for this route and high CH_4_ selectivity.^17,18^ Because methane selectivity is suppressed in the reaction facilitated by Pt–Co catalysts, this behavior also supports that Co is built into the system, and the increased catalytic activity arises from the electronic structure changes due to the alloy formation. Linking these findings with the results of the RWGS test reactions, we conclude that Pt–Co/MCF-17 catalysts exhibit “Pt-like” behavior with improved performance. This is consistent with other observations of “Pt-like” behavior and performance^24^ and proves that, by increasing the Co content from M-PtCo/MCF-17 to H-PtCo/MCF-17, the catalyst material still exhibits this behavior, with a decreased activity.

### 3.4 *Quasi In Situ* XPS

**Figure 6:**
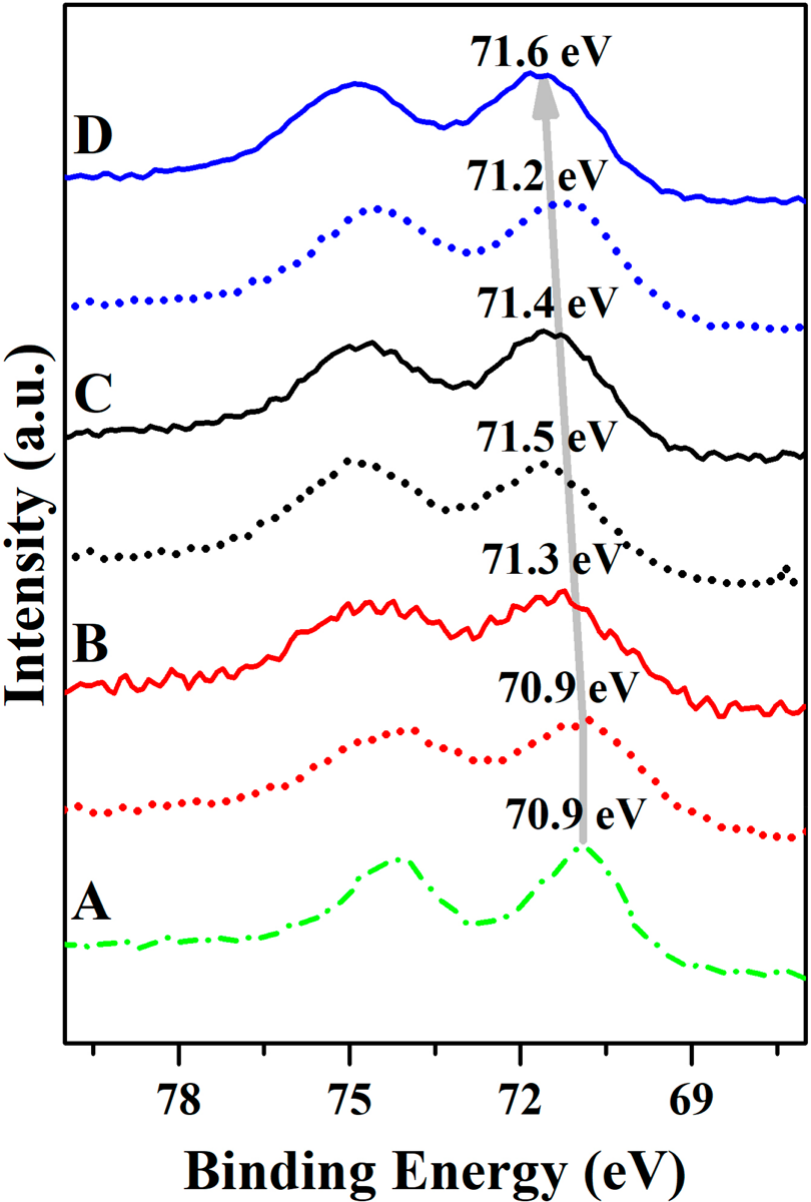
*Quasi in situ* XPS spectra of the Pt–Co/MCF-17 catalysts before and after being spent in CO_2_ hydrogenation: (A) 10-Pt/MCF-17; (B) 10-L-PtCo/MCF-17; (C) 10-M-PtCo/MCF-17; (D) 10-H-PtCo/MCF-17. The solid lines represent the spent state and the dotted lines the pretreated state of the Pt 4f transitions. The dash-dotted line of 10-Pt/MCF-17 represents the pure untreated Pt state as a benchmark.

For the *quasi in situ* XPS results, the peak-fitting procedure is discussed in the Supporting Information. Here we show the Pt 4f spectrum region of each Pt–Co alloy catalyst in the pretreated state and spent after an RWGS reaction. In both states, the binding energies of the detected Pt correspond to the Pt^0^ state and no platinum oxides were detected. As a reference, the binding energy of the Pt 4f_7/2_ peak of the 10-Pt/MCF-17 material was determined as 70.9 eV. In the case of pretreated 10-L-PtCo/MCF-17, the binding energy is the same, despite the presence of Co in the material. the When Co content is increased, 10-M-PtCo/MCF-17 and 10-H-PtCo/MCF-17 have 71.5 and 71.2 eV binding energies for the Pt 4f_7/2_ peak after pretreatment, respectively. As the literature suggests, Pt–Co alloy created by annealing bulk Pt(111) and a Co overlayer creates an alloy domain at the interface of the metals by dissolution of Co in Pt. This process yields a Pt 4f_7/2_ binding energy ranging from 71.6 to 71.4 eV.^53^ The spent catalyst materials 10-L-PtCo/MCF-17, 10-M-PtCo/MCF-17, and 10-H-PtCo/MCF-17 have Pt 4f_7/2_ peaks at 71.3, 71.4, and 71.6 eV binding energies, respectively; on the basis of this observation, we conclude that the binding energies of the spent catalysts shift to higher values, indicating the surface segregation of Pt atoms (Figure 6). This should be beneficial to the catalytic performance because the Pt atoms have higher electronegativity, resulting in the Co atoms donating electrons to the Pt atoms, which results in Pt atoms with local electron accumulation. This accumulation of electrons on the Pt atoms is enhanced on the tips and edges of the crystal structure, synergizing with the alloying effect.^25^ To interpret these changes, the surface energy of the metals should also be considered as an important factor because the HAADF images show that there are minor enrichments of the metals in the bimetallic Pt–Co NPs. Pt metal has a lower surface free energy of ∼2.490 J m^2–^, while creating a pure Co surface requires a higher energy investment of ∼2.540 J m^2–^.^54^ This is in agreement with the results published by Alayoglu et al.^24^ in that Pt will segregate to the surface in a reductive atmosphere (during pretreatment in H_2_ or RWGS reaction), preventing contact between the reactants and the Co-rich sites of the catalysts.

We also confirm that the particles are not embedded to the SiO_2_ structure because that would lead to increased plasmon features in the Pt 4f region and the standard metallic peak shape would not be eligible for the fit.^55^

### 3.5 Characterization of Spent Catalysts

**Figure 7:**
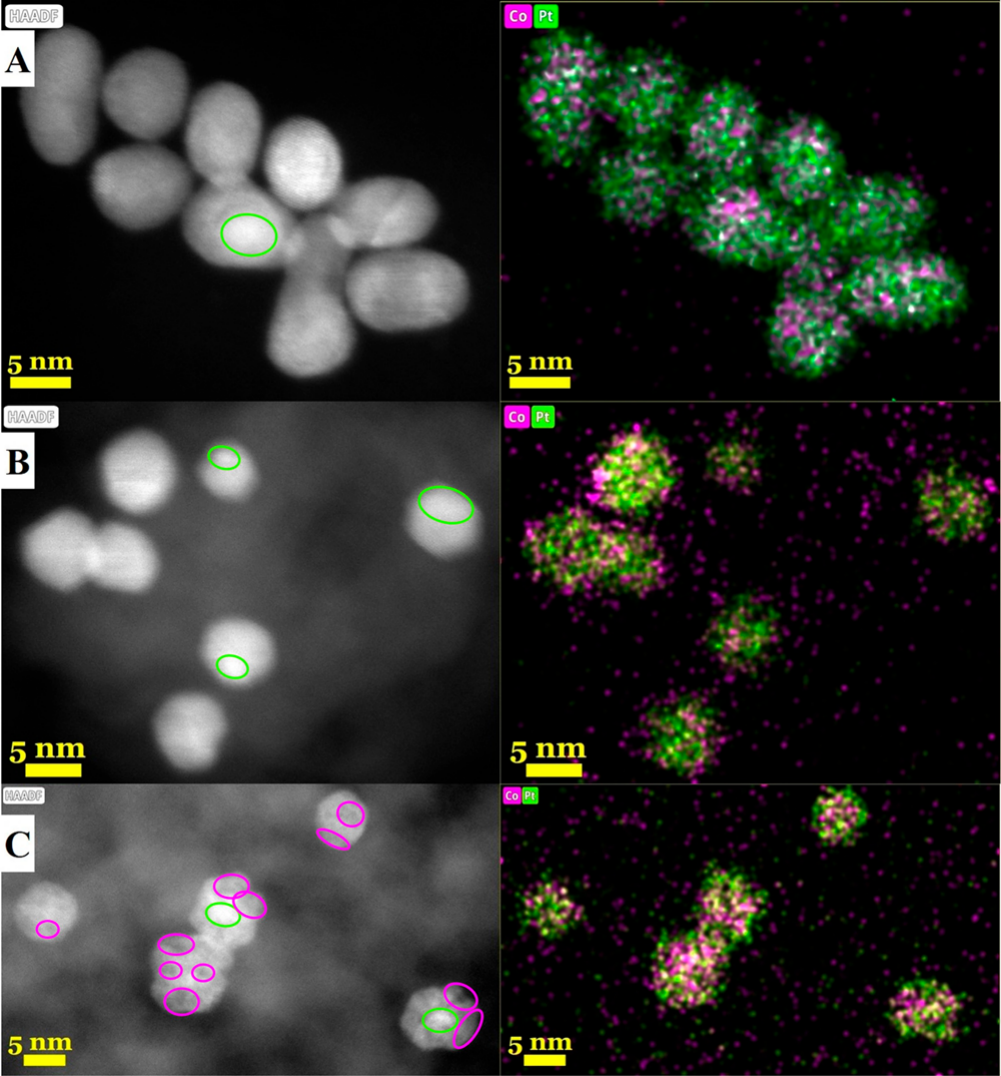
HAADF images and EDX element mapping of the Pt–Co/MCF-17 catalysts spent in atmospheric CO_2_ hydrogenation: (A) L-PtCo/MCF-17; (B) M-PtCo/MCF-17; (C) H-PtCo/MCF-17. Minor enrichments of Pt and Co are marked on the HAADF images, with circles matching the colors of the EDX element mapping.

To confirm any changes in the structure of the nanoparticles during the reaction, the spent catalysts were investigated with HR-TEM and HAADF (S)TEM with EDX. Figures S13 and S14 show that the nanoparticles maintain their dispersion, shape, and size and are not prone to sintering. Figure 7 demonstrates that the particles go through smaller rearrangements, but distinguished core–shell nanoparticles do not form with the surface segregation of Pt atoms. In the HAADF images, L-PtCo and M-PtCo particles show a homogeneous distribution after being spent in the RWGS reaction compared to the prepared state. However, EDX mapping shows a more intensive signal for Pt, which can be explained by a slight enrichment of Pt atoms in the outer atomic layers of the nanoparticles. H-PtCo particles still show minor enrichments, mainly of Co. While Pt has a lower surface free energy, Co, being in abundance, does not allow for Pt atoms to emerge and rearrange the alloy structure.

It is reported in the literature that annealing CoPt nanoclusters (*d* = 2–4 nm) at 600 °C under vacuum results in an increase of the *d*_111_ values by ∼1%, and this effect is due to local atomic relaxations.^56^ This phenomenon is expected to be crucial for the relative stability of nanoalloys or bimetallic nanostructures. We found that our nanoparticles did not go through this change of *d*_111_ (or a change in the *d* value for other Miller index planes) according to the values derived from the pattern of the Fourier transform (FT) HR-TEM images (Table S3), proving the high stability of the alloy structure during the RWGS reaction.

## 4 Summary and Conclusion

Bimetallic Pt–Co NPs of uniform average diameter and size distribution were synthesized and tuned by different ratios of Co metal (Pt:Co = 3.54, 1.51, and 0.96). Pure Pt NPs were also prepared as a benchmark material. The nanoparticles were supported on MCF-17 mesoporous silicon oxide, which produced high specific surface area catalysts (∼450 m^2^ g^–1^). The prepared materials were characterized with XRD, BET, TEM, HAADF (S)TEM, and EDX, revealing that Co atoms are built into the nanoparticles as an alloy structure. The catalysts were tested in a thermally induced (200–700 °C) RWGS reaction at atmospheric pressure. During test reactions, the Pt–Co bimetallic particles outperformed the pure Pt benchmark, and M-PtCo/MCF-17 showed the highest CO_2_ consumption and conversion over the given temperature range. At 500 °C, CO_2_ consumption was 2.6 times higher than that of Pt/MCF-17 or L-PtCo/MCF-17 catalysts and 1.7 times higher than that of H-PtCo/MCF-17. The Co-enhanced catalysts showed better (>98%) CO selectivity compared to the ∼95.1% achieved with the Pt benchmark, indicating that the presence of Co suppressed CH_4_ formation. This behavior was elucidated with the aid of *quasi in situ* XPS and *in situ* DRIFTS techniques. The changes in the Pt 4f binding energies measured by XPS can be attributed to the Pt atoms segregating from the sample surface. This process changes the electron configuration of the nanoparticles because electron accumulation on the Pt atoms is beneficial for higher catalytic activity. *In situ* DRIFTS indicated that all of the reactions on all of the catalysts take the formate reaction pathway, confirming the “Pt-like” behavior for L-PtCo/MCF-17, M-PtCo/MCF-17, and H-PtCo/MCF-17. By characterizing the spent samples with HAADF (S)TEM and EDX, we confirm that the particles are not prone to sintering but go through lesser rearrangement due to Pt segregating to the surface, as evidenced by XPS. These findings show that the electronic configuration is optimized for M-PtCo/MCF-17 when the molar ratio of Pt:Co is 1.51, and further increasing the Co content compromises the catalytic activity.

## Supporting Information Available

TEM images of the prepared materials (Figure S1) and prepared catalysts (Figure S2), high-resolution BF images and their FT patterns (Figure S3), comparison between the calculations and experimental observation of XRD (Figure S4), Pt and Co loadings of the catalysts (Table S1), BET isotherms of the catalysts (Figure S5) with derived data (Table S2), results of the catalytic stability tests (Figure S6), CO_2_ conversion (Figure S7) and product selectivity (Figure S8) of the catalytic test reactions, formation rate of products (Figure S9), aTOF values of the catalysts at 500 °C (Figure S10), *in situ* DRIFT spectra (Figure S11), example fit of the Pt 4f XPS spectra (Figure S12), BF TEM images of the spent catalysts (Figures S13 and S14), and table of data derived from the FT patterns of the BF HR-TEM images (Table S3) (PDF)
