# Supplementary material for: Optimized Pt–Co Alloy Nanoparticles for Reverse Water–Gas Shift Activation of CO2
Source: ACS Appl Nano Mater. 2024 Apr 24;7(9):9968–77. doi: 10.1021/acsanm.4c00111 (PMC11091851; doi:10.1021/acsanm.4c00111)
Supplement: Supplementary file 1 — an4c00111_si_001.pdf [file an4c00111_si_001.pdf]

## Supporting Information

# Optimized PtCo Alloy Nanoparticles for Reverse Water Gas Shift Activation of $CO_2$

**Ákos Szamosvölgyi[1], Ádám Pitó[1], Anastasiia Efremova[1], Kornélia Baán[1], Bence Kutus[2], Mutyala Suresh[†,1], András Sápi[1]*, Imre Szenti[1,3], János Kiss[1,3], Tamás Kolonits[4], Zsolt Fogarassy[4], Béla Pécz[4], Ákos Kukovecz[1], Zoltán Kónya[1,3]**

[1]*University of Szeged, Interdisciplinary Excellence Centre, Department of Applied and Environmental Chemistry, H-6720, Rerrich Béla tér 1, Szeged, Hungary*

[2]*University of Szeged, Department of Molecular and Analytical Chemistry, H-6720, Dóm tér 7-8, Szeged, Hungary*

[3]*University of Szeged, Institute of Environmental and Technological Sciences, H-6720, Szeged, Hungary*

[4]*University of Szeged, HUN-REN-SZTE Reaction Kinetics and Surface Chemistry Research Group, H-6720, Szeged, Hungary*

[5]*HUN-REN Centre for Energy Research, Institute of Technical Physics and Materials Science, H-1121, Budapest, Hungary*

*Corresponding author at the University of Szeged, Interdisciplinary Excellence Centre, Department of Applied and Environmental Chemistry, H-6720, Rerrich Béla tér 1, Szeged, Hungary.

E-mail address: sapia@chem.u-szeged.hu (A. Sápi).

## S1. TEM images

Bright-field TEM images of the prepared materials.

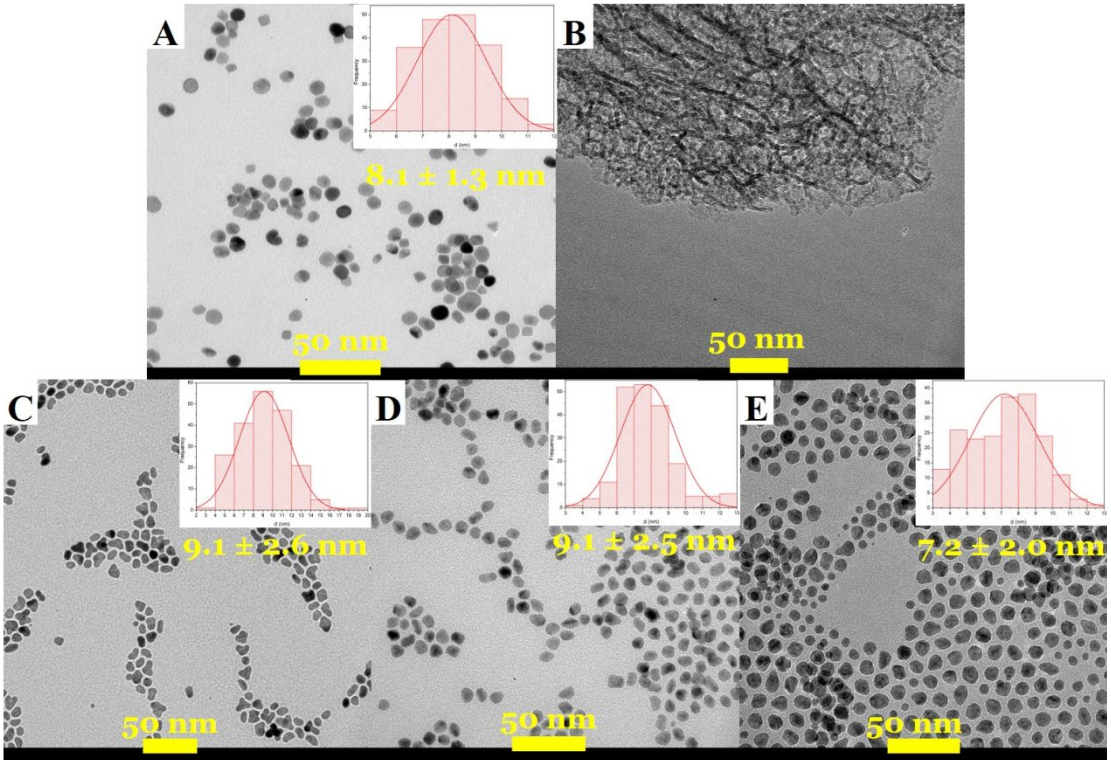


**Fig. S1.** TEM images of the synthesized A) Pt nanoparticles, B) MCF-17 support material, C) L-PtCo, D) M-PtCo, and E) H-PtCo nanoparticles

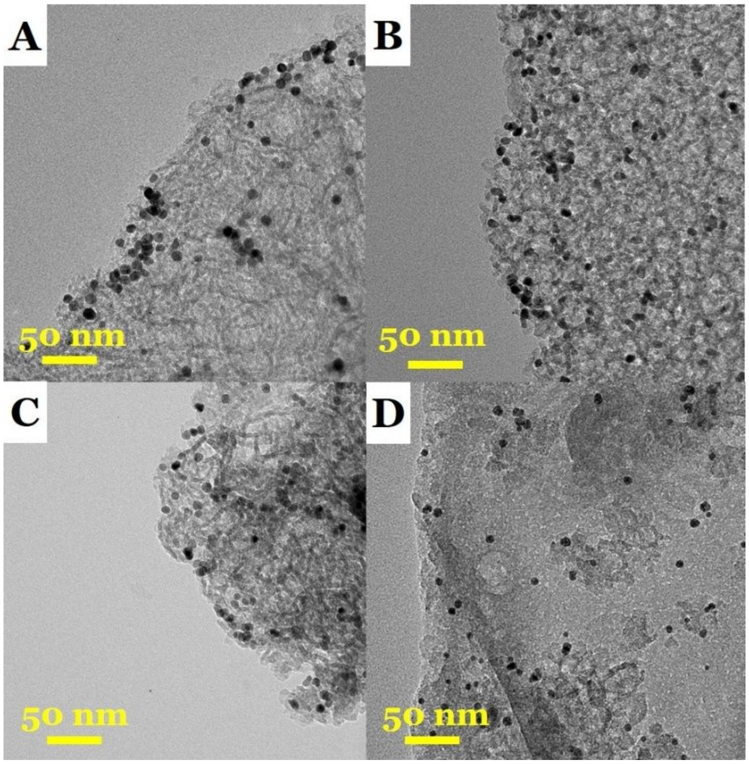


**Fig. S2.** TEM images of the catalysts prepared from the MCF-17 support material and the nanoparticles: A) Pt/MCF-17, B) L-PtCo/MCF-17, C) M-PtCo/MCF-17, D) H-PtCo/MCF-17

The crystal planes identified from the patterns of the Fourier transform HR TEM images (Fig. S3) agree with the XRD diffractograms. The wide, blurry rings are due to the amorphous MCF-17 $SiO_2$.

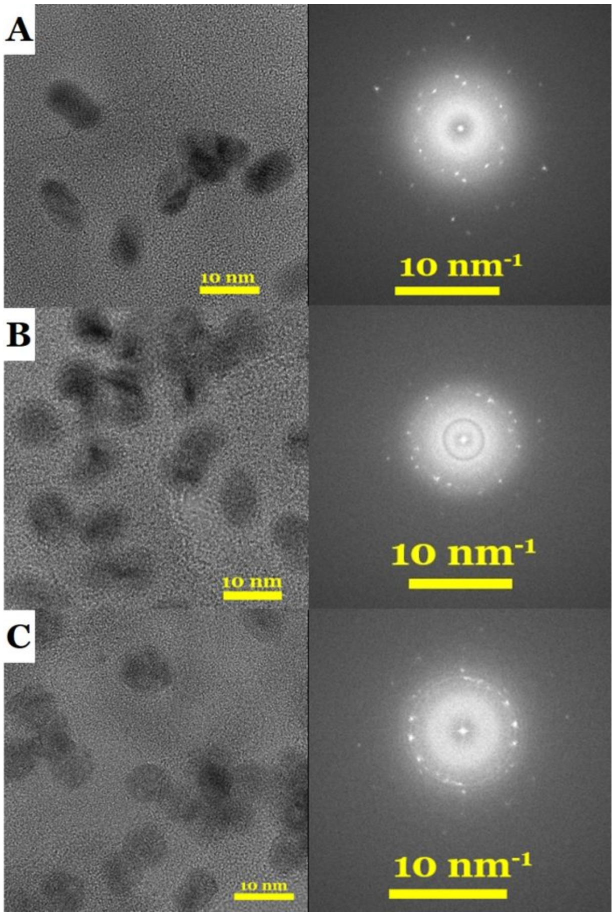


**Fig. S3.** HR BF images and their Fourier transform patterns of the prepared bimetallic catalysts: A) L-PtCo/MCF-17, B) M-PtCo/MCF-17, C) H-PtCo/MCF-17.

## S3. XRD

The data for calculated reflections of the Pt, $CoPt_3$, and CoPt were derived from the Materials Project database [1]. The comparison with the experimentally observed data is shown below in Fig. S4.

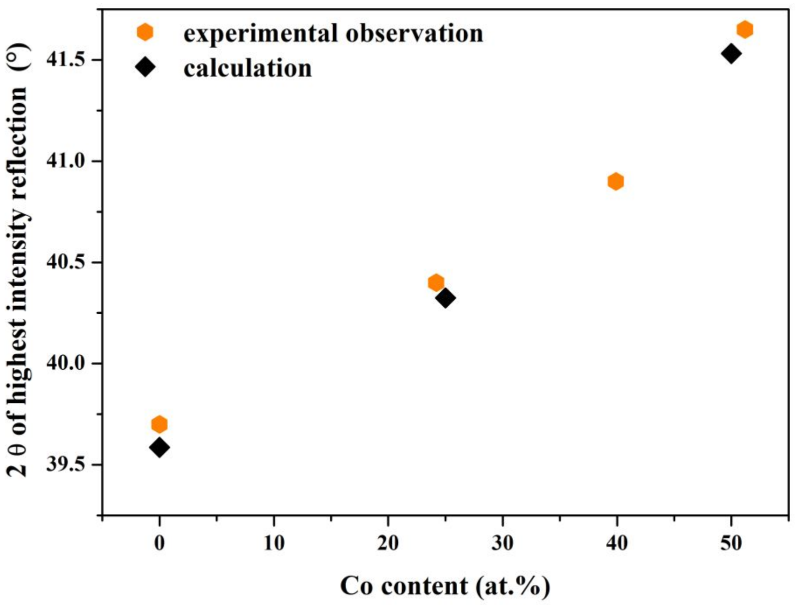


**Fig. S4.** Comparison between the synthesized materials and the calculated data by the shifts of the (111) Miller-index plane as the function of Co content in the synthesized nanoparticles

## S2. ICP-MS

After applying the nanoparticles to the MCF-17 support material, the elemental composition of the materials was analyzed with the ICP-MS technique. Table S1 shows the nominal and the measured metal concentrations of the prepared catalysts.

**Table S1.** Pt and Co loadings of the investigated catalysts confirmed by ICP- MS

| Catalyst | ICP-MS analysis | | | |
|---|---|---|---|---|
| | Nominal Pt:Co ratio | Measured Pt:Co ratio | Pt (w/w %) | Co (w/w %) |
| Pt/MCF-17 | - | - | 0.97 | 0 |
| L-PtCo/MCF-17 | 3 | 3.54 | 0.70 | 0.06 |
| M-PtCo/MCF-17 | 1 | 1.51 | 0.56 | 0.11 |
| H-PtCo/MCF-17 | 0.5 | 0.96 | 0.51 | 0.16 |

## S3. Nitrogen adsorption and desorption

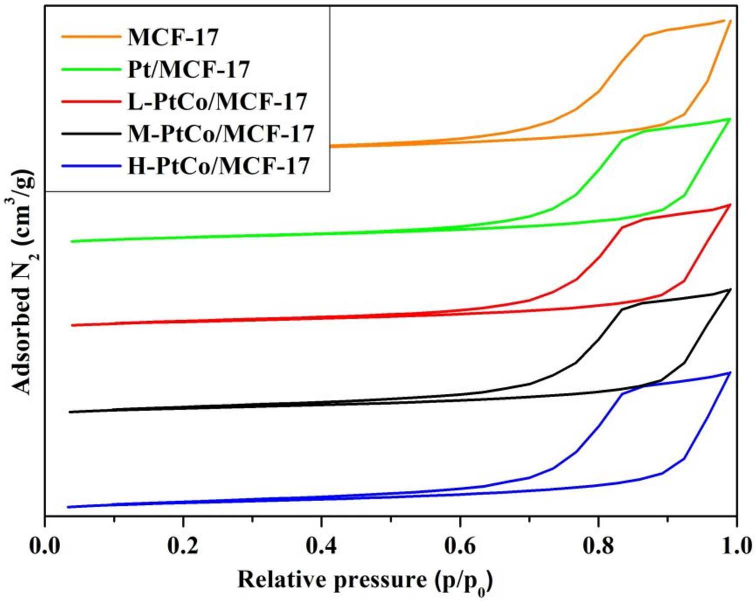


**Fig. S5.** The $N_2$ adsorption and desorption isotherms of the prepared catalysts

The plotted data shows a type IV isotherm with a hysteresis loop for all the Pt-Co alloy and Pt catalysts. By the steepness of the isotherms, it is evident our materials have relatively uniform channel-like pores or a pore network (H2 type hysteresis) [2]. Specific surface areas were calculated based on the Brunauer-Emmett-Teller theory and equation.

**Table S2.** Summarized data from the nitrogen adsorption-desorption experiments

| | Specific surface area ($m^2$/g) | Total pore volume ($cm^3$/g) | Average pore size diameter (nm) |
|---|---|---|---|
| MCF-17 | 581 | 1.34 | 9.2 |
| Pt/MCF-17 | 472 | 1.46 | 12.4 |
| L-PtCo/MCF-17 | 416 | 1.42 | 13.7 |
| M-PtCo/MCF-17 | 451 | 1.49 | 13.2 |
| H-PtCo/MCF-17 | 471 | 1.52 | 12.9 |

## S4. RWGS test reaction

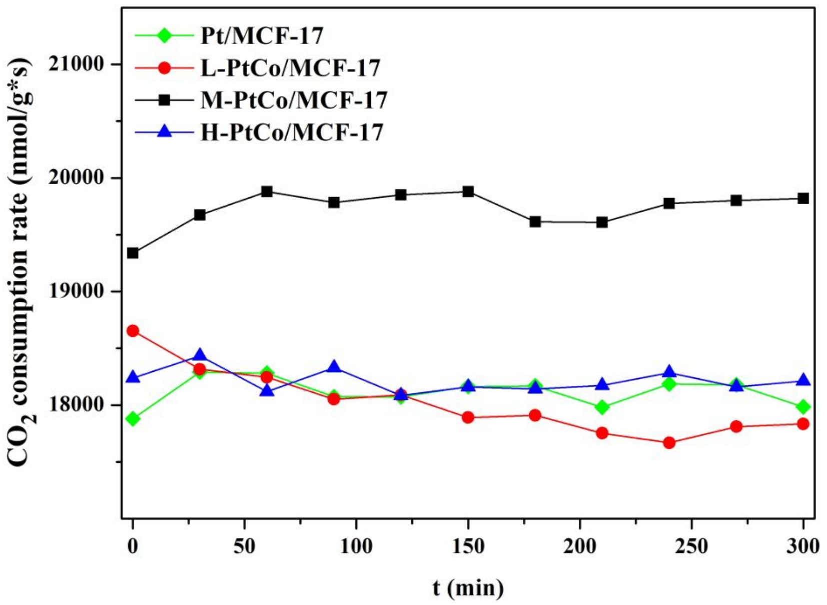


**Fig S6.** Proof of catalytic stability of the MCF-17 supported Pt-Co based catalysts during RWGS reaction, isotherm conditions over 6 hours after reaching 700 °C reaction temperature, with the same reaction conditions as described in the main article

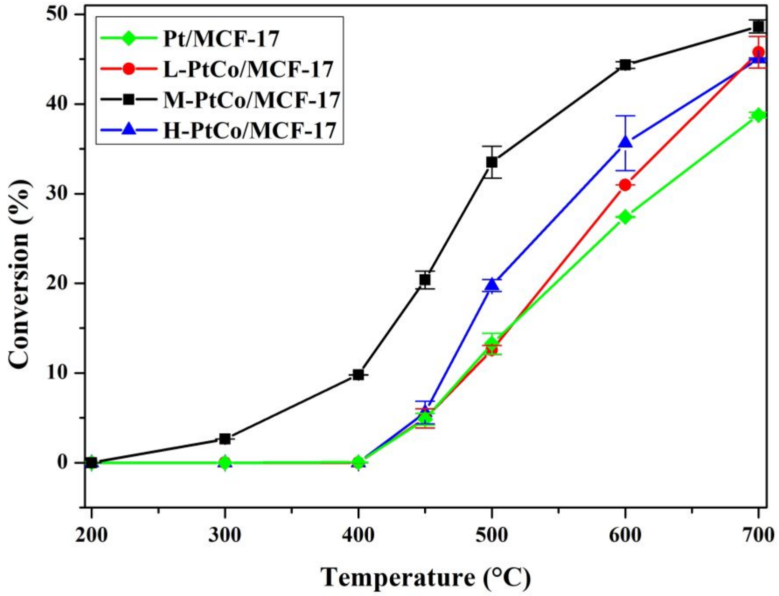


**Fig. S7.** Conversion of $CO_2$ during RWGS reaction as the function of temperature

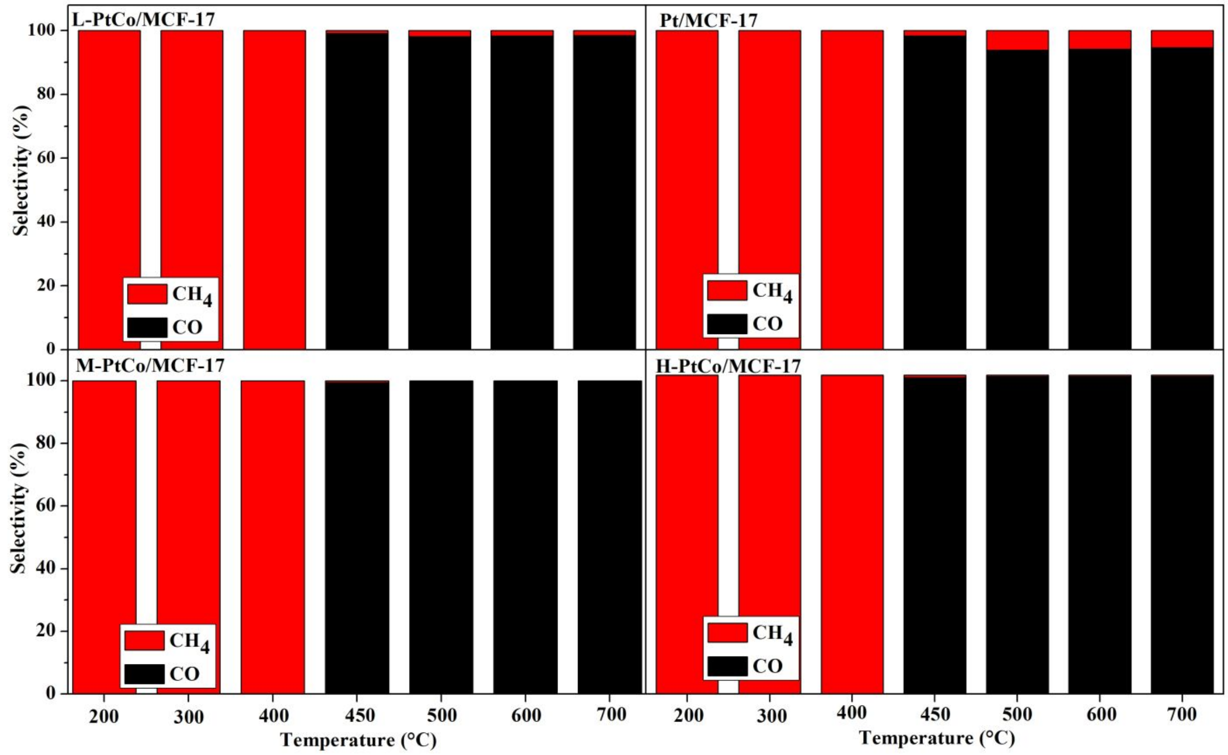


**Fig. S8.** Product selectivity of each tested catalyst on all analyzed temperature steps

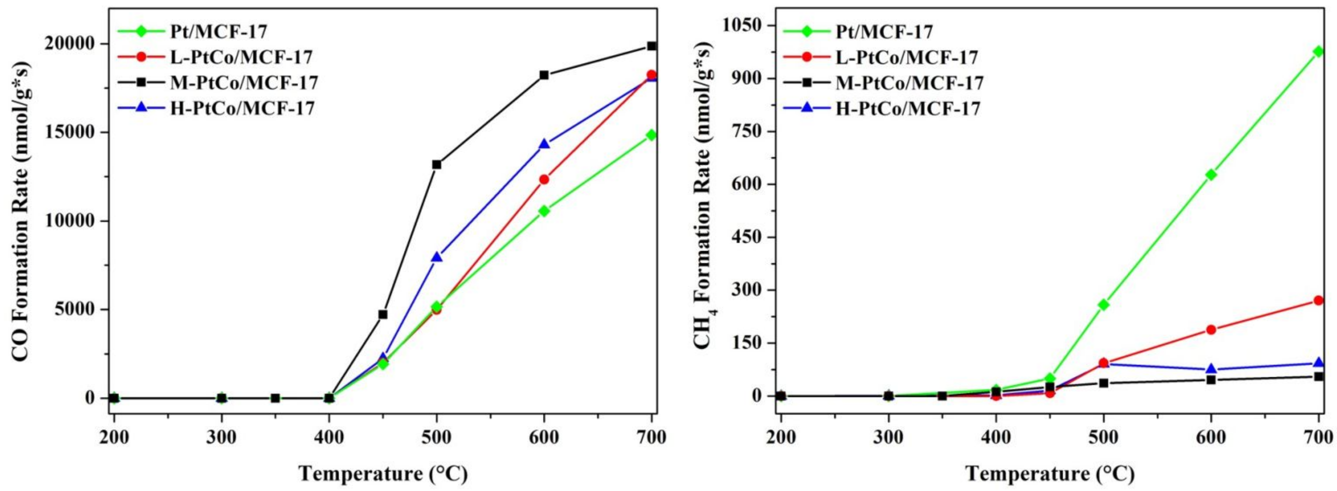


**Fig. S9.** CO formation rate (left) and $CH_4$ formation rate (right) in RWGS reaction of Pt, and Pt-Co nanoparticles supported on MCF-17

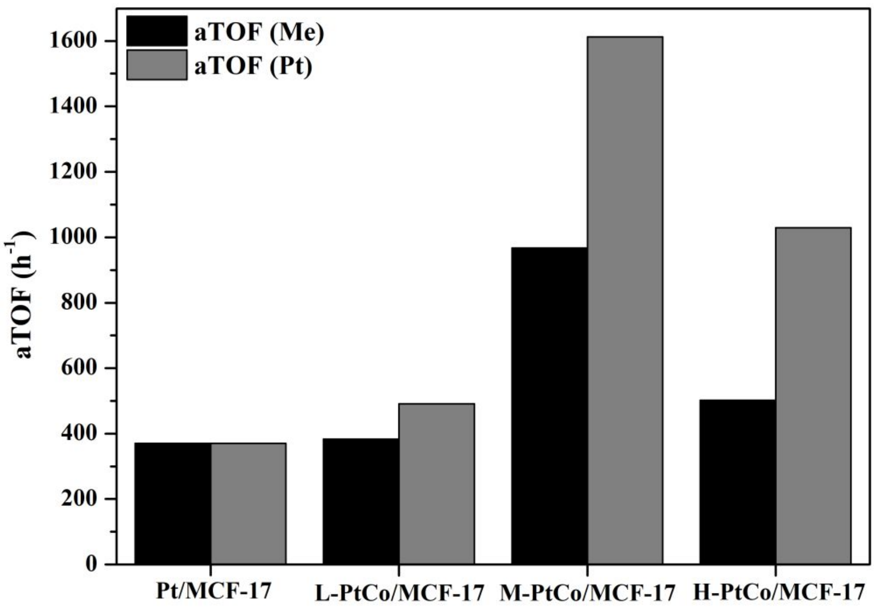


**Fig S10.** aTOF values of the tested PtCo catalysts at 500 °C calculated from the quantity of $CO_2$ converted specified for the loading of metal atoms

## S5. *In situ* DRIFTS

In situ DRIFTS measurement of the Pt/MCF-17 and Pt-Co/MCF-17 samples with all the relevant temperature steps.

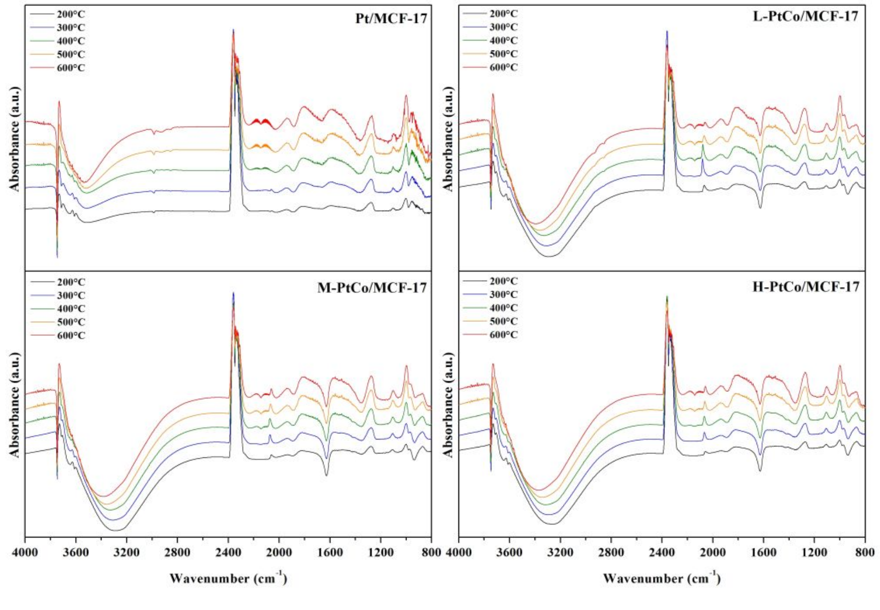

**Fig.S11** *In situ* DRIFT spectra recorded during RWGS reaction from 200 to 600 °C catalyzed by the Pt and Pt-Co alloy nanoparticle – MCF-17 materials

## S6. *Quasi in situ* XPS data evaluation

The acquired data was evaluated using Casa XPS software version 2.3.5PR1.0 [3]. All high-resolution spectra were corrected with a Shirley background and all peaks were fit with a Gauss-Lorentzian product function, where the Lorentzian contribution was 30%. The aliphatic component of the C 1s spectra at 284.8 eV was used as an inner Binding Energy reference. All Binding Energy values are relevant to 0.1 eVs. Pt 4f peaks were fit with an asymmetric Lorentzian (LA) peak shape LA(1.2, 85, 70) [4]. An example of the fit is presented below in Fig. S9.

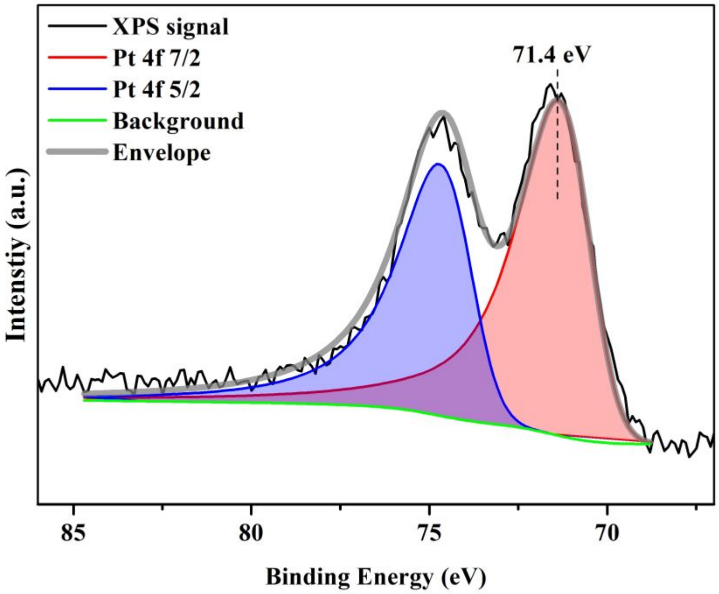


**Fig S12.** Example for the fit of *quasi in situ* XPS Pt 4f regions with asymmetric peak shape for the elemental platinum detected in the case of spent 10-M-PtCo/MCF-17 catalyst

## S7. TEM images of the spent catalysts

The Pt and Pt-Co nanoparticles supported by MCF-17 were investigated with TEM once again after they were spent in RWGS reaction to verify there was no particle aggregation/sintering during the reaction.

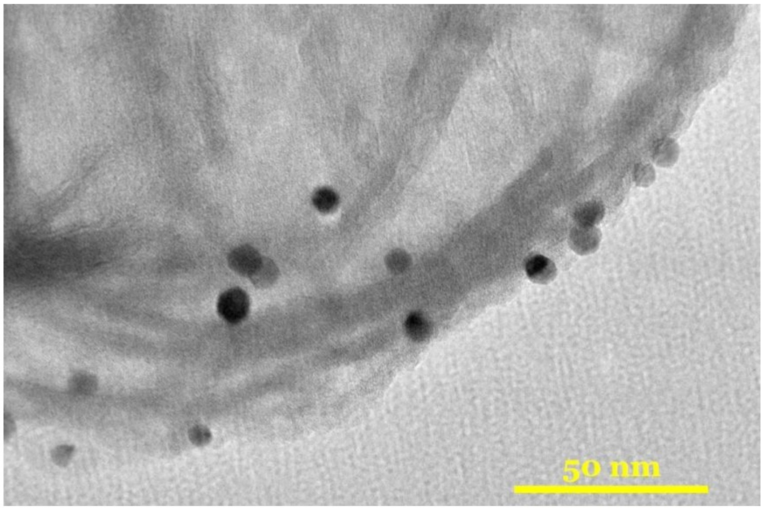


**Fig. S13.** BF TEM image of spent the Pt/MCF-17 catalyst

Patterns of Fourier transform HR TEM images (Fig S11) still show the same lattice d values, thus the primer crystallites remain in the fcc crystal structure.

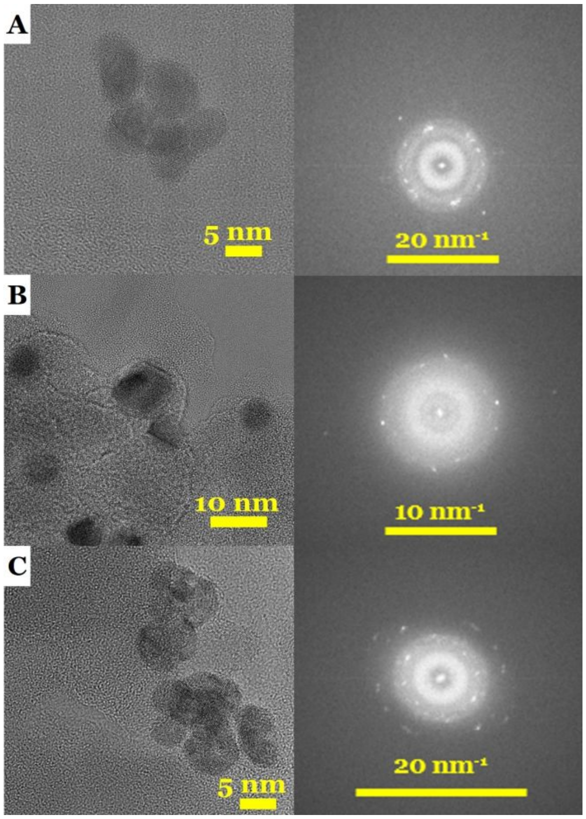


**Fig. S14.** HR BF images and patterns generated by Fourier transform of the spent catalysts: A) L-PtCo/MCF-17, B) M-PtCo/MCF-17, C) H-PtCo/MCF-17.

**Table S3.** Data derived from the Fourier transform of the BF HR TEM images; lattice parameter (d) and interplanar spacing of the Miller-index planes characteristic to the Pt fcc crystal structure

| Sample | d [nm] | (111) | (200) | (220) | (311) | (222) | (400) |
|---|---|---|---|---|---|---|---|
| L-PtCo/MCF-17 | 0.3829 | 220 | 192 | 135 | 116 | 110 | 96 |
| M-PtCo/MCF-17 | 0.3758 | 217 | 187 | 133 | N/A | 108 | N/A |
| H-PtCo/MCF-17 | 0.3772 | 217 | 189 | 135 | N/A | 109 | N/A |
| Spent L-PtCo/MCF-17 | 0.3802 | 219 | 190 | 134 | N/A | 113 | N/A |
| Spent M-PtCo/MCF-17 | 0.3773 | 215 | 190 | 132 | N/A | 110 | 94 |
| Spent H-PtCo/MCF-17 | 0.3796 | 218 | N/A | 134 | 116 | 109 | N/A |

## References


1. Jain, A.; Ong, S. P.; Hautier, G.; Chen, W.; Richards, W. D.; Dacek, S.; Cholia, S.; Gunter, D.; Skinner, D.; Ceder, G.; Persson, K. A., Commentary: The Materials Project: A materials genome approach to accelerating materials innovation. *APL Materials* **2013,** *1* (1), 011002.
2. Sing, K. S. W., Reporting physisorption data for gas/solid systems with special reference to the determination of surface area and porosity (Recommendations 1984). *Pure and Applied Chemistry* **1985,** *57* (4), 603-619.
3. Fairley, N.; Fernandez, V.; Richard ‐ Plouet, M.; Guillot-Deudon, C.; Walton, J.; Smith, E.; Flahaut, D.; Greiner, M.; Biesinger, M.; Tougaard, S.; Morgan, D.; Baltrusaitis, J., Systematic and collaborative approach to problem solving using X-ray photoelectron spectroscopy. *Applied Surface Science Advances* **2021,** *5*, 100112.
4. Su, H.; Ye, Y.; Lee, K.-J.; Zeng, J.; Mun, B. S.; Crumlin, E. J., Probing the surface chemistry for reverse water gas shift reaction on Pt(111) using ambient pressure X-ray photoelectron spectroscopy. *Journal of Catalysis* **2020,** *391*, 123-131.
